# Removal of the innocuity test from *The International Pharmacopoeia* and WHO recommendations for vaccines and biological products^☆^

**DOI:** 10.1016/j.biologicals.2020.05.003

**Published:** 2020-07

**Authors:** Dianliang Lei, Herbert Schmidt, Ivana Knezevic, Tiequn Zhou, Hye-na Kang, Sabine Kopp

**Affiliations:** Unit of Technical Specifications and Standards, Department of Health Product Policy and Standards, Division of Access to Medicines and Health Products, World Health Organization, Geneva, Switzerland

**Keywords:** International standard, Innocuity test, Biologicals, Vaccines, Pharmaceuticals, Removal

## Abstract

The innocuity test was indicated as a quality control test to release pharmaceutical and biological products to the market. The test was intended to detect possible extraneous toxic contaminants derived from the manufacturing processes of the product. The test was included in WHO Recommendations and Guidelines for vaccines, biotherapeutics and blood products and in some monographs on antibiotics in *The International Pharmacopoeia*. Over the past years, the requirements in WHO Recommendations/Guidelines for conducting the test evolved such that it could be waived for routine release of product once consistency of production was established to the satisfaction of the NRA, or that the need for this test should be discussed and agreed with the NRA. However, some users of WHO written standards for biologicals (i.e., Recommendations, Guidelines) and WHO specifications for pharmaceuticals (i.e*., The International Pharmacopoeia*) requested that the innocuity test be deleted from WHO written standards based on its lack of specificity and scientific relevance. In response to that request, we studied the history of this test and its use by the member states of WHO, and the recommendations in WHO written standards. The outcomes of the study were reviewed by the relevant WHO Expert Committee on Biological Standardization and Expert Committee on Specifications for Pharmaceutical Products who then decided to discontinue this test in WHO Recommendations for vaccines and biologicals and to omit the test from *The International Pharmacopoeia*.

## 1 Introduction

The innocuity test (also referred to as the abnormal toxicity test, general safety test or undue toxicity test) was originally developed in the early 1900s to ensure the safe and consistent production of serum products containing phenol-derived preservatives [1]. The test was later expanded to a general safety test to detect possible extraneous toxic contaminants (other than, for example, bacterial endotoxins) in biological products [1]. The principle of the test consists of injecting the product into guinea pigs and/or mice. A batch passes the test if no animal shows any signs of illness, relevant body weight changes, or dies within 7 days. The exact test protocol and interpretation of the outcome can vary significantly between different national pharmacopoeias or requirements [1]. In WHO written standards including Recommendations and Guidelines for vaccines (http://www.who.int/biologicals/en), blood products (http://www.who.int/bloodproducts/en/), and *The International Pharmacopoeia* (https://www.who.int/medicines/publications/pharmacopoeia/en/) this test was included as a safety test for the finished final product. Despite significant advances in analytical techniques, method validation approaches, manufacturing processes and quality control, this test was retained with the intention to detect possible product/process toxic contaminants and to avoid batch-to-batch differences in quality [1].

In recent years, however, the value of the test has been questioned – both from the perspective of regulatory science and in the context of the principles of animal use. There have been many debates on the rationale and value of the test and its inherent limitations. In particular, there were concerns on its lack of specificity, poor reproducibility and its scientific relevance was questioned in global meetings and publications. In recent decades the implementation of Good Manufacturing Practices (GMP), the use of validated manufacturing processes as well as appropriate and more stringent quality control measures in production of pharmaceutical and biological products have rendered its use as questionable [2,3]. In 2016 the 17th International Conference of Drug Regulatory Authorities (ICDRA) held in South Africa, with participance of most of national regulatory authorities in the world, made recommendations to WHO to consider removal of the innocuity test as a requirement for lot release from WHO vaccine guidelines but encourage maintenance of some capacity to perform this test, if needed [4]. In light of this, some national regulatory authorities indicated that the omission of the innocuity test would not compromise the safety of medicines within their jurisdictions [5,6]. In 2015, the International Alliance for Biological Standardization (IABS) and the European Partnership for Alternative Approaches to Animal Testing (EPAA) each held international conferences at which the utility of the innocuity test was debated. The meeting participants made concrete recommendations to overcome existing barriers to the global deletion of innocuity test or general safety tests, from requirements for human biological products and from veterinary vaccines [7]. Requests to WHO to initiate steps to delete the innocuity test from all WHO's written standards were made based by some WHO member states and other stakeholders.

## 2 **WHO's perspectives** on safety and potency testing

WHO's position on the innocuity test has evolved over the past years to reflect the changes to requirements in various national regulations (e.g. Pharmacopoeia), and supported by scientific evidence. WHO's perspectives on safety test and potency testing was reviewed in 2012 and outlined that WHO has supported the concept of replacement, reduction and refinement in use of animals for developing, producing, testing and characterizing vaccines for human use [8]. The innocuity test was required for lot release on each final lot of the vaccines in WHO recommendations/guidelines developed before 1999 (http://www.who.int/biologicals/en), such as the first Requirement for pertussis vaccine [9] and Requirements for diphtheria toxoid and tetanus toxoid [10]. However, since the year 2000, the requirements within WHO guidelines for conducting the test were made more flexible. For example, in the Recommendations for *Haemophilus influenzae* type b conjugate vaccines it was indicated that the test may be omitted for routine lot release once consistency of production has been well established to the satisfaction of the national regulatory authority and when good manufacturing practices are in place” [11]. In Guidelines for typhoid conjugate vaccines [12] and Recommendations for inactivated poliomyelitis vaccines [13] the recommendation has been changed to as “*The need to test the final lots of the vaccine for unexpected toxicity (also known as abnormal toxicity) should be agreed with the NRA. This test may be omitted from routine lot release once the consistency of production has been established to the satisfaction of the NRA, and when reliable GMP are in place*”.

The history of recommending the innocuity test in the manufacture of biotherapeutic and blood products resembles that of vaccines; where the test was required in earlier Recommendations and Guidelines, this was relaxed in later documents with deferral to national requirements. For example, the Recommendations for blood products state that “*the abnormal toxicity test is still required by some pharmacopoeias and is performed at the stage of product development. However, because of the very limited quality control value of this assay, it is increasingly being abandoned by most regulatory authorities. Correct implementation of GMP should provide evidence that the product would comply with the test for abnormal toxicity*” [14].

At the sixty-seventh meeting of the Expert Committee on Biological Standardization (ECBS) in 2016, the Committee responded to the request from a regulatory agency to delete the innocuity test from all WHO Recommendations, Guidelines and other guidance documents:

“The Committee noted that deletion of the abnormal toxicity test from the European Pharmacopoeia will provide additional impetus for the global elimination of this test, although this may take some time to achieve. It was pointed out that the US Code of Federal Register had already taken this step and was no longer requiring the General Safety test. Furthermore, the recently revised WHO Recommendations for HPV vaccines, adopted by the Committee in 2015, included the note in small print that some countries no longer require this test. Further consideration of this issue will be required by the Committee at its future meetings.” [15].

Considering the varying level of implementation of GMP in WHO Member States, the ECBS decided that national regulatory authorities should decide at their own discretion whether the innocuity test is needed to release a vaccine or not [15]. However, the innocuity test was still listed in the control tests of final products of WHO documents.

Also, in *The International Pharmacopoeia,* published by WHO, the test for undue toxicity test was used to determine the absence of undue toxicity of antibiotics intended for parenteral administration” and applied to kanamycin acid sulfate and kanamycin monosulfate [16].

## 3 Decisions made by the national regulatory authorities and pharmacopoeias

A number of national regulatory authorities and Pharmacopoeias have already removed, or are working on the removing of the innocuity test from their requirements or pharmacopoeias. In 2015, the U.S.A. Food and Drug Administration (FDA) published in the Federal Register their intent to delete the innocuity (general safety) test:

“The Food and Drug Administration (FDA) amending the biologics regulations by removing the general safety test (GST) requirements for biological products. FDA is finalizing this action because the existing codified GST regulations are duplicative of requirements that are also specified in biologics license applications (BLAs), or are no longer necessary or appropriate to help ensure the safety, purity, and potency of licensed biological products. FDA is taking this action as part of its retrospective review of its regulations to promote improvement and innovation, in response to the Executive order.” [6].

This rule became effective August 3, 2015; however, it was not automatically applied to pre-existing BLAs or BLA supplements. Manufacturers would need to submit applications to the FDA for each biological license in order to discontinue or modify the test according to the new regulation. Until approval of the individual applications, the general safety test would continue to be required for all biological products [6].

During the 159th plenary session of the European Pharmacopoeia Commission, held in Strasbourg on 21–22 November 2017, the Commission endorsed the complete suppression of the test for abnormal toxicity from the European Pharmacopoeia [5]. As part of this exercise, the test for abnormal toxicity was removed from 49 monographs, including 36 monographs on vaccines for human use. In addition, as the general chapter Abnormal Toxicity (2.6.9 of Ph. Eur.) was no longer referenced in any monograph, it was subsequently rendered obsolete and was also deleted from the Ph. Eur. The suppression of the test for abnormal toxicity became effective on January 01, 2019 [5].

Health Canada also decided to remove the abnormal toxicity test from its requirements for medicines as required in its Post-Notice of Compliance [17]. In Brazil, the abnormal toxicity test is no longer required for vaccines. Manufacturers may stop performing the test for licensed products after communication to the Brazilian regulatory authority. The Argentinian regulatory authority has removed innocuity test from the Pharmacopoeia for biologicals. In South Africa, the National Control Laboratory has eliminated the innocuity test from routine testing of vaccines for the purpose of lot release [18].

In India, the test was required by the regulatory authority, but after the introduction of GMP it was recommended that, for certain products including vaccines, the innocuity test may be omitted for routine lot release once the consistency in production has been well established and approved by the national regulatory authority. The test is still required for newly developed products until the consistency in production is established to the satisfaction of NRA. For established products, the vaccine manufacturers shall apply for the waiver as a post-approval change for omission of the test [19].

In China, Japan and Russia, innocuity testing is still required in their Pharmacopoeia or Minimum Requirements for Biological Products as a safety test for the release of vaccines and biological products [18,20,21].

## 4 Decision of Expert Committees of WHO

Following the recommendation of ECBS in 2016, WHO secretariat reviewed the requirements for this test in regulations of its member states and in WHO published Recommendations and Guidelines. A report of the study was presented to the ECBS Committee at its meeting in 2018. The ECBS reviewed the development and the current use of innocuity testing for the purpose of marketing authorization or lot release. The test has historically been included in WHO Recommendations and Guidelines from the onset and in national pharmacopoeias worldwide. The Committee reviewed the requests from regulators, industry and other agencies to WHO to initiate steps to delete the innocuity test from all WHO Recommendations, Guidelines and other WHO written standards. The scientific rationale and evidence for performing the innocuity test as a measure of the safety of vaccines and other biological products for the purpose of marketing authorization and lot release were discussed in depth by the Committee. Current manufacturing processes, which include the implementation of GMP and comprehensive quality control measures (including in-process controls), were considered to be more appropriate than the innocuity test in assuring the quality and safety of vaccines and other biological products. The Committee then reviewed the historical inclusion of the innocuity test in documents published in the WHO *Technical Report Series* and concluded that its complete omission would not compromise the quality and safety of vaccines and other biological products. As a result, the Committee recommended the immediate discontinuation of the inclusion of the innocuity test in all future WHO Recommendations, Guidelines and manuals for biological products published in the *Technical Report Series*. The Committee further recommended that the inclusion of this test in previously published WHO *Technical Report Series* documents be disregarded. It was considered that these recommendations represented a significant step towards science-based regulation and regulatory convergence at the global level. This decision was published in ECBS report in WHO *Technical Report* Series, No. 1016 in 2018 [22].

Following the decision of ECBS, the Expert Committee on Specifications for Pharmaceutical Products (ECSPP) also considered a proposal to remove the test for undue toxicity (Chapter 3.7) from *The International Pharmacopoeia*, including the references to the test in the monographs on Kanamycin acid sulfate and Kanamycin monosulfate. The proposal was discussed at the informal consultation on Screening Technologies and Pharmacopoeial Specifications for Medicines in May 2019, and sent out for public consultation from August to October 2019. At its meeting in October 2019, the Committee agreed to omit the test for undue toxicity (Chapter 3.7) from *The International Pharmacopoeia* [23]. The reference to the test for undue toxicity in the monographs on kanamycin acid sulfate and kanamycin monosulfate will be removed from *The International Pharmacopoeia* in its next of the 10th Edition [24].

## 5 Conclusion

The WHO Expert Committee on Biological Standardization and the WHO Expert Committee on Specifications for Pharmaceutical Products discontinued the recommendation of the innocuity test/test for undue toxicity in quality specifications for finished pharmaceutical and biological products. The test is no longer required for the purpose of WHO prequalification of vaccines and medicines for UN supply. The deletion of the innocuity test/test for undue toxicity is also in line with the replacement, reduction, and refinement (3Rs) initiative.
